# Optimization of media and temperature for enhanced antimicrobial production by bacteria associated with *Rhabditis* sp.

**Published:** 2013-06

**Authors:** Siji Jinachandrannair Vijayakumari, Nishanth Kumar Sasidharannair, Bala Nambisan, Chellappan Mohandas

**Affiliations:** Central Tuber Crops Research Institute, Thiruvananthapuram – 695 017, Kerala, India

**Keywords:** Antimicrobial activity, Entomopathogenic nematodes, Symbiotic bacteria, Bioactive molecules

## Abstract

**Background and Objectives:**

Entomopathogenic nematodes, belonging to the family heterorhabditis and steinernematidae, are reported to be symbiotically associated with specific bacteria and the secondary metabolites produced by these bacteria possess antimicrobial activity. In this study, bacteria were isolated from nematodes belonging to the family rhabditidae, and the antimicrobial activity was tested against four bacteria viz. *Bacillus subtilis* MTCC 2756, *Staphylococcus aureus* MTCC 902, *Escherichia coli* MTCC 2622, and *Pseudomonas aeruginosa* MTCC 2642 and five fungi viz. *Aspergillus flavus* MTCC 183, *Candida albicans* MTCC 277, *Fusarium oxysporum* MTCC 284, *Rhizoctonia solani* MTCC 4634 and *Penicillium expansum* MTCC 2006.

**Materials and Methods:**

The isolated bacteria were cultured in nutrient broth (NB), Luria broth (LB) and Tryptic soya broth (TSB) at 25, 30 and 35°C. Cell free culture filtrate was prepared by centrifugation and was separated into organic and aqueous fractions. Organic fraction was concentrated and tested for antimicrobial activity.

**Results:**

The culture filtrate of the bacteria isolated from the entomopathogenic *Rhabditis* sp. was found to possess antimicrobial activity against the four bacteria and five fungi tested. The bacterium grew well in TSB, LB and NB media though in TSB yield and activity were higher. Antimicrobial activity was higher at 30°C as compared with 25 or 35°C. HPLC analysis indicated major differences in peak areas and retention times at different temperatures. Increased number of peaks with higher peak areas was obtained at 30°C.

**Conclusion:**

The study suggests that the bacteria could produce more bioactive molecules effective against medically and agriculturally important bacteria and fungi depending on culture media and temperature. Modified media could yield different types of molecules effective against diseases/disorders of plant, animals and humans.

## INTRODUCTION

*Xenorhabdus* and *Photorhabdus*, symbiotic bacteria, associated with *Steinernema and Heterorhabditis* respectively, have been reported to produce many secondary metabolites having wide range of bioactivities of medicinal and agricultural interest such as antibiotic, antimycotic, insecticidal, nematicidal, antiulcer, antineoplastic and antiviral (1). The infective juveniles of the entomopathogenic nematodes (EPN) enter through natural orifices such as the mouth, anus or spiracles and release the bacteria into the hemocoel and both kill the host rapidly within 48h. A new EPN was isolated from sweet potato weevil grubs from the Central Tuber Crops Research Institute farm, which belonged to the genus *Rhabditis* and subgenus *Oscheius* (rhabditidae: nematoda). It was found to behave like the EPN by having specific symbiotic bacteria pathogenic to insects (2) and could be isolated from 3^rd^ stage infective juveniles of the nematode, from hemolymph of nematode infested *Galleria mellonella* larvae and from a number of insects parasitized by the nematode. The bacterium has been found to have 99% similarity to *Bacillus cereus* strain 03BB102 (Accession No. CP001407) based on 16S rDNA sequencing and blast analysis in NCBI (3).

*Xenorhabdus* and *Photorhabdus* produce several compounds exhibiting antibacterial and antifungal activity, many of which have been identified from the bacterial cultures. Some of these include compounds such as nematophins (indole derivatives), xenorhabdins (dithiolopyrrlones), xenocoumacins (benzopyran-1-one derivatives) and anthraquinones (4–7). The present study is carried out with the objective of evaluating the antimicrobial activity of the bacterial symbiont isolated from rhabditid nematode and to optimize conditions for maximum activity.

## MATERIALS AND METHODS

### Preparation of culture filtrate

The bacteria isolated from *Rhabditis (Oscheius)* sp. was cultured in nutrient broth (NB), Luria broth (LB) and Tryptic soya broth (TSB) to find the best nutrient medium for antibiotic production. Growth of the bacteria was studied in the three media at different time intervals (24, 48, 72, 96, 120, 144 and 170 h.). One loopfull of bacterial culture was transferred into 100 ml of different sterile medium in 250 ml flasks. The flasks were incubated in the dark at 30°C on a rotary shaker at 150 rpm. When the optical density at 600 nm was approximately 1.7, the bacterial cultures were transferred under aseptic conditions into 1 L flasks, containing 400 ml sterile media. The flasks were then incubated in the dark at 30°C on a rotary shaker at 150 rpm. Following fermentation, the cultures were centrifuged at 10000 rpm for 15 minutes at 4°C and passed through 0.4µm bacterial filters to obtain the cell free culture broth (8).

### Separation of culture filtrates into organic and aqueous fractions

TSB, LB and NB culture filtrates (1.5 L) were further processed for separation into aqueous and organic fractions. The filtrate was neutralized with concentrated hydrochloric acid and extracted with an equal volume of ethyl acetate thrice. The ethyl acetate layers were combined, dried over anhydrous sodium sulphate, and concentrated using a rotary flash evaporator at 30°C. The dry residue was weighed and reconstituted in methanol and used for assay of antimicrobial activity and HPLC analysis.

### Antimicrobial Assay

Antibacterial activity of the crude fractions was measured using agar diffusion assays (9) against Gram positive (*Bacillus subtilis* MTCC 2756, *Staphylococcus aureus* MTCC 902) and Gram negative bacteria (*Escherichia coli* MTCC 2622, and *Pseudomonas aeruginosa* MTCC 2642). Commercial antibiotics ceftazidime (30 µg/ml) and Ciprofloxacin (5 µg/ml) were used as positive reference standard. Antibacterial activity was evaluated by measuring the diameter of the inhibition zone. Methanol controls were also tested along with the test samples.

Antifungal activity was determined by agar disc diffusion method (10) against medically important (*Aspergillus flavus* MTCC 183, *Candida albicans* MTCC 277) and agriculturally important fungi (*Fusarium oxysporum* MTCC 284, *Rhizoctonia solani* MTCC 4634 and *Penicillium expansum)* by measuring diameter of zones of inhibition. Amphotericin was used as control for *C. albicans* whereas bavistin (100 µg/ml) was used for the remaining four fungi.

### High performance liquid chromatography (HPLC) analysis

The concentrated ethyl acetate fractions of TSB culture filtrate at different temperatures (25, 30 and 35°C) were dissolved in methanol, filtered through 0.2 µm filters, and subjected to HPLC (C18 column, 250 x 4.6, flow rate 1ml/min, UV detection at 200 nm) using methanol: water (50:50) as mobile phase.

### Statistical analysis

Statistical analysis of the data was done using SPSS 17.0. Means were compared using One-way ANOVA of Duncan test.

## RESULTS

### Optimization of growth conditions

**Fig. 1:**
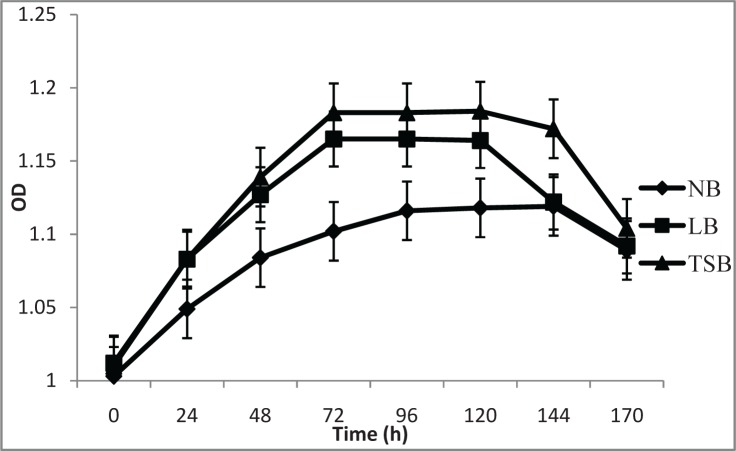
Bacterial growth on NB, LB and TSB broths at different time intervals.

The effect of three different culture media on bacterial growth was studied. Bacteria showed strong, exponential growth up to 48 h. and reached stationary phase at 96 h. (Fig. 1).

**Fig. 2:**
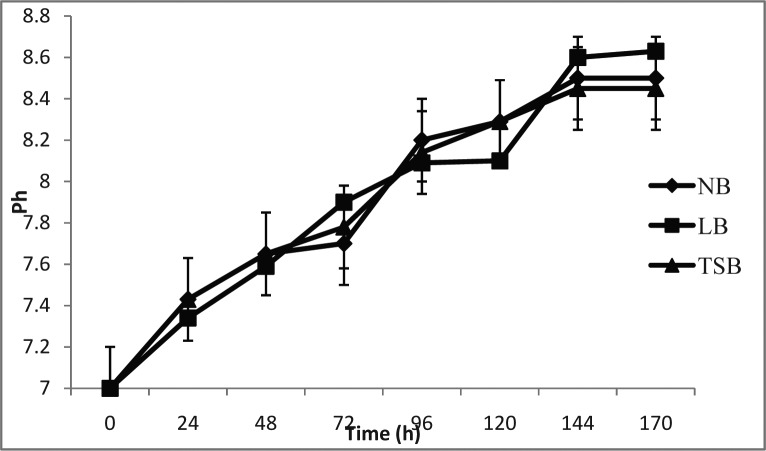
pH change on NB, LB and TSB broths at different time intervals.

The pH of the bacterial culture increased steadily from 7.0 to 8.8 upto 144 h. and then remained constant (Fig. 2). The same pattern was observed in all three media. Bacterial growth was relatively higher in TSB medium.

### Separation of culture filtrates into organic and aqueous fractions

The culture filtrates were extracted with ethyl acetate to obtain an organic fraction which was concentrated and weighed. Maximum yield was obtained in TSB medium (0.64 g) followed by LB (0.58 g) and NB (0.23 g) per 1.5 L culture broth.

### Antimicrobial activity

**Fig. 3:**
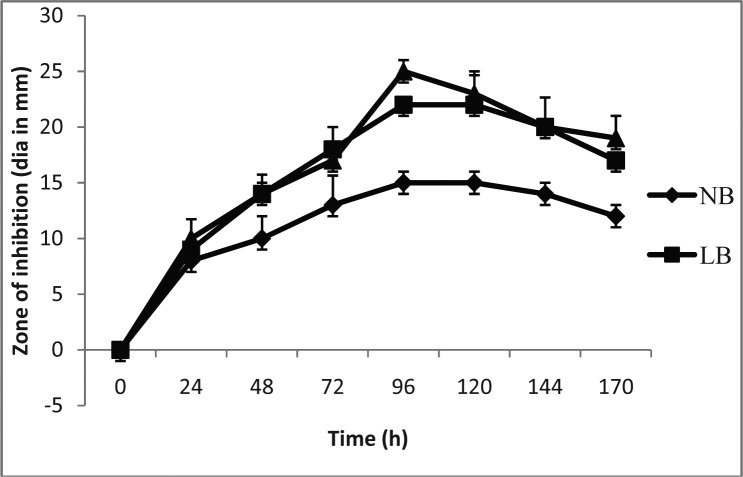
Antimicrobial activity of NB, LB and TSB broths against *Bacillus subtilis*.

The ethyl acetate fractions were tested for antibacterial activity against the four pathogenic bacteria. In all three media, activity was detectable from the 24 h. which reached maximum level at 96 h. Against *B. subtilis* 15, 17 and 26 mm diameter zones were recorded in NB, LB and TSB respectively at 96 h. (Fig. 3).

The crude NB extract had no activity against *S. aureus* and *P. aeruginosa* (Table 1).

**Table 1:** Antibacterial activity of organic fraction.

Test bacteria	Diameter of zone of inhibition (mm)				

	NB	LB	TSB	Ceftotaxime	Ciprofloxacin
*B. subtilis*	15^a^	17^b^	26^c^	-	31
*S. aureus*	-	31 ^b^	35 ^c^	-	31
*E. coli*	14^a^	17 ^b^	23 ^c^	-	28
*P. aeruginosa*	-	28 ^b^	32 ^c^	-	25

Values followed by the different letters in the same row are significantly different (p < 0.05) - no zone of inhibition

Antifungal activity of the fractions was tested against the five fungi. TSB extract recorded significantly higher activity against the five test fungi, compared to the other two extracts (Table 2). The antifungal activity was nearly comparable to that of the standard fungicide Bavistin.

**Table 2:** Antifungal activity of crude extract.

Test fungi	Diameter of zone of inhibition (mm)				

	NB	LB	TSB	Bavistin	Amphotericin
*C. albicans*	10^a^	30 ^b^	35 ^c^	-	23
*A. flavus*	9 ^a^	15 ^b^	31 ^c^	25	-
*F. oxysporum*	12 ^a^	20 ^b^	30 ^c^	16	-
*R. solani*	7 ^a^	17 ^b^	22 ^c^	19	-
*P. expansum*	17 ^a^	45 ^b^	49 ^c^	24	-

Values followed by the different letters in the same row are significantly different (p < 0.05) - not tested

### Effect of the temperature on yield and activity

**Fig. 4:**
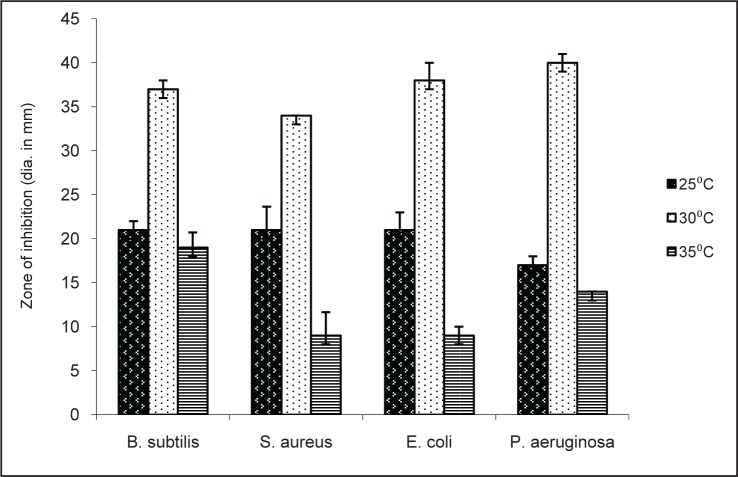
Antibacterial activity of TSB extract at different temperatures.

**Fig. 5:**
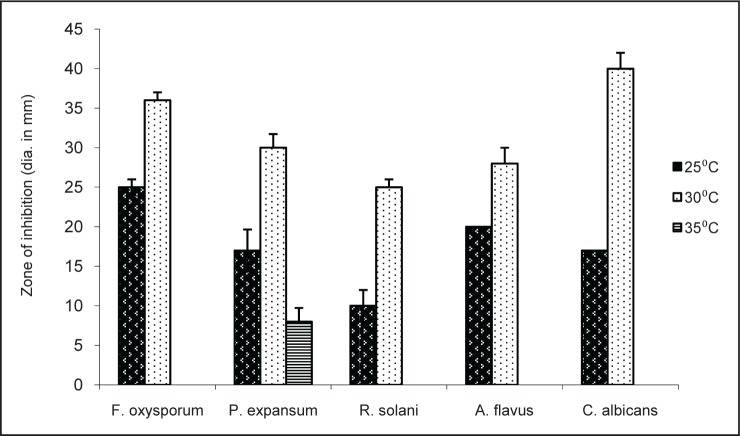
Antifungal activity of TSB extract at different temperatures.

As the TSB culture medium produced highest antimicrobial activity the effect of temperature on activity was tested by carrying out the fermentation at 25, 30 and 35°C. Ethyl acetate fractions were tested for antibacterial and antifungal activity. Maximum antibacterial and antifungal activity was observed at 30°C. (Figs. 4, 5). At 35°C antifungal activity was completely lost except against *P. expansum*.

### HPLC profile

**Fig. 6:**
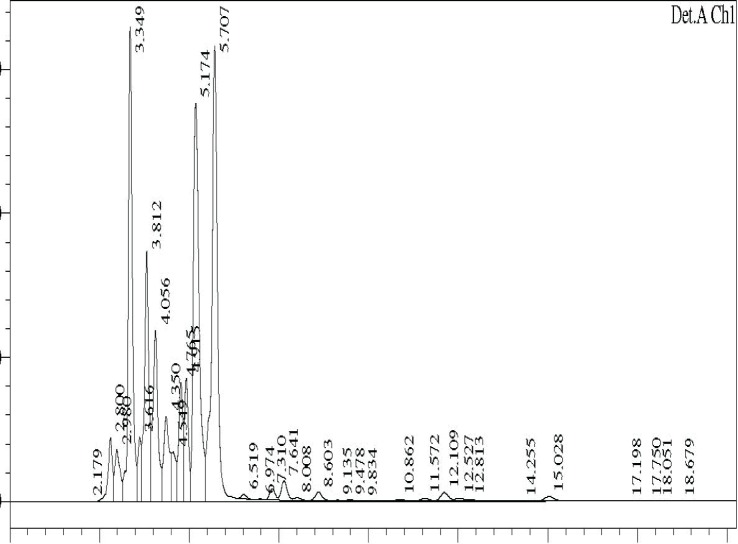
HPLC profile of TSB extract at 25°C.

**Fig. 7:**
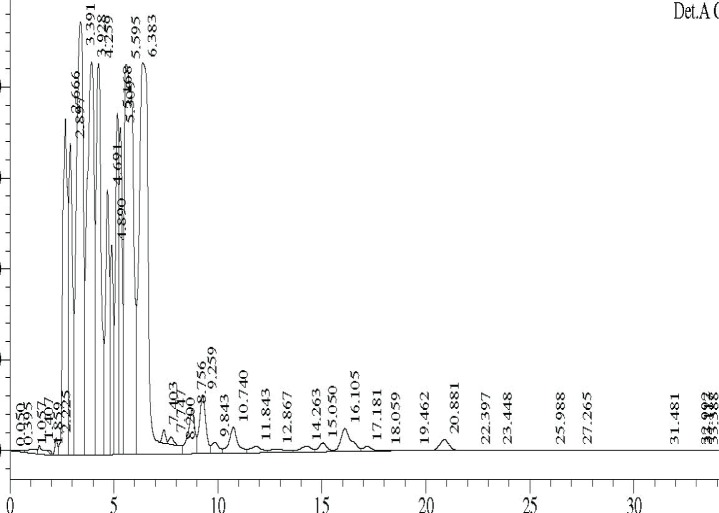
HPLC profile of TSB extract at 30°C.

**Fig. 8:**
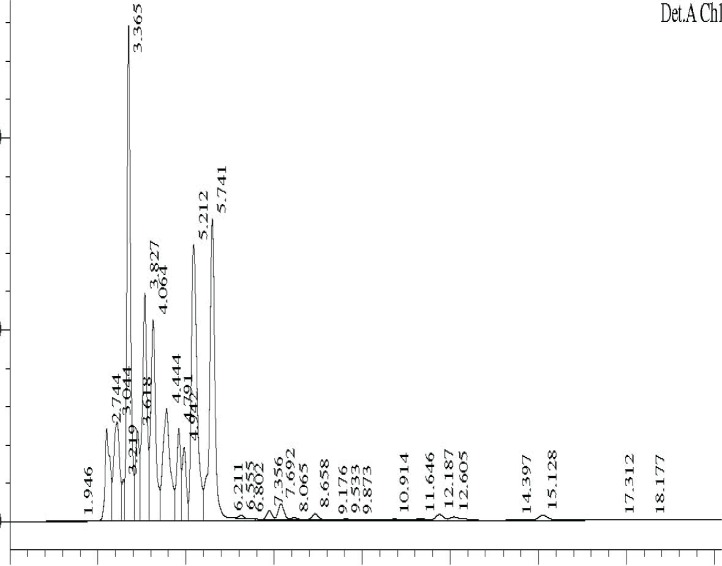
HPLC profile of TSB extract at 35°C.

The ethyl acetate fractions obtained from TSB culture filtrates at different temperatures were analysed by HPLC. The profiles are shown in (Figs. 6–8).

Major differences were observed in the three profiles with respect to peak area and retention times (Table 3).

**Table 3:** Area and retention time of major peaks in HPLC profiles of TSB extracts at different temperature.

Retention time of major peaks (min)	Area of peak*		
	30°C	25°C	35°C
2.66	29.6	-	-
2.89	21.0	1.8	1.5
3.39	54.5	13.1	4.64
3.80	-	7.3	2.72
4.06	-	5.8	2.56
4.26	37.1	-	-
4.40	-	3.2	2.15
4.69	18.1	-	-
4.89	11.8	3.3	-
5.17	21.5	15.6	4.17
5.30	17.5	-	-
5.60	64.6	18.5	4.54
6.38	68.1	-	-

* area x10^6^

Comparison of peaks having similar retention times showed that the peak area was significantly higher in the 30°C samples, followed by 25°C and 35°C samples, suggesting the presence of higher concentration of active compounds at 30°C. The data on the HPLC profiles suggest that the compounds produced by fermentation at different temperatures could be different.

## DISCUSSION

The present study relates to a new bacterial symbiont associated with a novel EPN belonging to the *Rhabditis (Oscheius)* sp. The nematode–bacterium combination showed pathogenicity against insect pests, similar to the association of *Xenorhabdus* and *Photorhabdus* with *Steinernema* and *Heterorhabditis*, respectively. A common characteristic of EPN bacteria is the production of antibacterial and antifungal metabolites. The culture filtrate of the new bacterium showed significant antimicrobial activity against several test organisms (bacteria and fungi). In our study TSB was found to be the best medium for bacterial growth, yield and bioactivity as compared to Luria broth and nutrient broth. The fermentation temperature also affected the biological activity of the extracts. Maximum yield and activity was obtained when the bacterial fermentation was carried out at 30°C. Earlier studies on the EPN bacteria *X. nematophilia* TB showed that cell growth and accumulation of metabolic products in cultures were strongly influenced by growth medium and fermentation conditions such as inoculum age, inoculation volume, pH, temperature and fermentation time (11, 12). Lower antibiotic activity was reported in *X. nematophilia* D at fermentation temperatures of 35°C than at 15 -30°C (13). This is in agreement with our results on the new bacterium.

Antibiotic production by the symbiotic bacterium *X. nematophilus* has been reported to differ qualitatively and quantitatively depending on the strain and species of bacteria and their culture conditions (14). Production of antibiotics by biocontrol agents in liquid culture can be affected by several factors such as pH, temperature and composition of the culture medium (15). The variation in the HPLC profiles in extracts obtained at different temperatures could possibly be attributed to the presence of different metabolites, which in turn influence antimicrobial activity. Since manipulation of the culture conditions can improve yield and bioactivity, there is room for further enhancement of production of antimicrobials by this novel bacterial symbiont isolated from the rhabdtid nematode.
